# Complex aortic plaques: hidden danger in aortic stenosis. Role of transesophageal echocardiography

**DOI:** 10.47487/apcyccv.v5i2.377

**Published:** 2024-06-24

**Authors:** Lindsay Benites-Yshpilco, Kelly Cupe-Chacalcaje, Angela Cachicatari-Beltrán, Josh Moscoso, Kevin Velarde-Acosta, Alessio Demarini-Orellana, Gerald Lévano-Pachas, Roberto Baltodano-Arellano

**Affiliations:** 1 Departamento de Cardiología Clínica, Hospital Guillermo Almenara Irigoyen - EsSalud, Lima, Peru. Departamento de Cardiología Clínica Hospital Guillermo Almenara Irigoyen - EsSalud Lima Peru; 2 Servicio de Cardiología, Área de Imagen Cardíaca, Hospital Guillermo Almenara Irigoyen - EsSalud, Lima, Peru. Servicio de Cardiología, Área de Imagen Cardíaca Hospital Guillermo Almenara Irigoyen - EsSalud Lima Peru; 3 Universidad de San Martín de Porres, Lima, Peru. Universidad de San Martín de Porres Universidad de San Martín de Porres Lima Peru; 4 Facultad de Medicina, Universidad Nacional Mayor de San Marcos, Lima, Peru. Universidad Nacional Mayor de San Marcos Facultad de Medicina Universidad Nacional Mayor de San Marcos Lima Peru

**Keywords:** Plaque, Atherosclerotic, Thoracic Aorta, Aortic stenosis, Echocardiography, Transesophageal, Placa Aterosclerótica, Aorta Torácica, Estenosis Aórtica, Ecocardiografía Transesofágica

## Abstract

Aortic stenosis is associated with aortic plaques in up to 85% of cases because they share risk factors and pathogenic pathways. Intrinsically, complex aortic plaques carry a high risk of stroke, which has also been demonstrated in the context of aortic stenosis, especially in patients who underwent percutaneous or surgical replacement. Transesophageal echocardiography (TEE) is the imaging test of choice to detect plaques in the thoracic aorta and classify them as complex plaques. Furthermore, the 3D modality allows us to better specify its dimensions and anatomical characteristics, such as added thrombi or the presence of ulcers inside. This review aims to evaluate the use of TEE to detect complex aortic plaques in patients with an indication for percutaneous or surgical aortic valve replacement. To highlight the association between aortic stenosis and complex aortic plaques, we attached to the review some TEE studies from our experience.

## RESUMEN

La estenosis aórtica se asocia con placas aórticas hasta en un 85% de los casos, porque comparten factores de riesgo y vías patogénicas. Intrínsecamente, las placas aórticas complejas conllevan un alto riesgo de accidente cerebrovascular, lo que también se ha demostrado en el contexto de estenosis aórtica, especialmente en pacientes sometidos a reemplazo percutáneo o quirúrgico. La ecocardiografía transesofágica (ETE) es la prueba de imagen de elección para detectar placas en la aorta torácica y clasificarlas como placas complejas. Además, la modalidad 3D nos permite precisar mejor sus dimensiones y características anatómicas como trombos añadidos o la presencia de úlceras en su interior. Esta revisión tiene como objetivo evaluar el uso de ETE para detectar placas aórticas complejas en pacientes con indicación de reemplazo valvular aórtico percutáneo o quirúrgico. Para resaltar la asociación de estenosis aórtica y placas aórticas complejas adjuntamos a la revisión algunos estudios de ETE de nuestra experiencia.

## Introduction

Aortic stenosis (AS) is the most common acquired valve disease affecting around 2% of adults over 65 years of age, with a higher prevalence due to aging populations ^1^^,^^2^. Narrowing of the aortic valve has serious clinical consequences and impacts survival in the absence of percutaneous or surgical valve replacement ^3^^,^^4^. On the other hand, atherosclerosis of the thoracic aorta is a degenerative process associated with aging; this is clinically relevant because it is a source of systemic embolization including stroke, transient ischemic attack (TIA), and peripheral embolization ^5^^-^^8^. In the last decades, transesophageal echocardiography has been crucial to its detection, structural characterization, and severity determination ^9^^,^^10^.

Previous research studies demonstrated the coexistence of degenerative AS and complex plaques in the thoracic aorta, which is explained by their shared risk factors and pathogenic mechanisms ^11^^,^^12^. This document is intended to provide the foundations of this association, its relationship with stroke, and describe the transesophageal echocardiography (TEE) in the study of aortic plaques. Finally, we add some images that demonstrate the usefulness of 2D/3D TEE in the study of aortic plaques.

### Pathophysiology

The pathophysiological mechanism of AS was long considered a passive degenerative process; however, it is currently known as a dynamic-molecular process that shares pathogenic pathways with atherosclerosis ^13^^,^^14^. Both have as the initial event the endothelial dysfunction, which favors tissue infiltration of low-density lipoproteins (LDLs) and lipoprotein A through endothelial cells with the consequent immune cell extravasation ^15^.

Increased oxidative stress promotes the formation of oxidized LDL, which triggers the vascular and valve inflammatory response, activating local macrophages, CD4+, CD8+ lymphocytes, T lymphocytes, and mast cells (16). After the inflammatory phase, the propagation stage will predominantly be in the valve tissue, in which cytokines secreted by immune cells promote the differentiation of valve interstitial cells into myofibroblasts and osteoblastic phenotypes, and this process will finally cause the diffuse release of calcium in the valve tissue ^16^^,^^17^.

### Association´s evidence and stroke risk 

The association between AS and aortic plaques/atheromas has been demonstrated in research studies of valvular patients undergoing TEE. Up to 85% of AS cases coexist with plaques in the thoracic aorta, which are thicker than in screening controls, and almost half are defined as complex atheromas (47%) ^18^. Furthermore, in an observational study, plaques were found to be distributed mainly in the descending aorta and in the aortic arch (77% and 66% of cases, respectively), while plaques in the ascending aorta were only present in 4% of patients with AS ^18^.

On the other hand, it has been shown that the presence of calcium in the aortic valve is the most powerful predictor of plaques in the aorta and is related to greater dimensions and complexity ^19^. In relation to severity, the majority of patients with severe AS had severe aortic atherosclerosis (54%), with an odds ratio for this condition of 4.9 compared to patients without AS ^20^. This trend was confirmed when the complex atheroma variable was used, in which patients with moderate to severe stenosis had more complex atheromas than those with mild stenosis ^21^. Conversely, a previous study reported that the presence of complex aortic plaque is an independent predictor of rapid progression of AS (0.41 m/s/year) and cardiac events ^22^.

Notably, the most striking characteristic of plaques in the thoracic aorta (including the descending aorta) is their high predictive value for cardiovascular diseases, embolic events, and mortality ^5^^,^^6^^,^^23^^,^^24^. Furthermore, when the plaques are complex, they have a much stronger association with stroke, reaching an odds ratio of up to 17.1 ^7^. Likewise, in the context of AS, two retrospective studies demonstrated that complex plaques in the aortic arch detected by TEE were associated with stroke with an odds ratio of 4.9 and 8.46 ^20^^,^^25^.

Additionally, given that patients with AS undergo cardiac catheterization procedures for diagnostic and/or therapeutic purposes, there is a potential risk of injuring the aortic plaque, causing cerebral or distal embolic events ^25^^-^^28^. Finally, during surgical replacement, aortic arch atheromas can increase the risk of stroke by 6 times and the risk of in-hospital mortality after cardiac surgery up to 2 times ^29^^,^^30^.

### Usefulness of transesophageal echocardiography

#### Aortic stenosis

In the majority of patients with AS, transthoracic echocardiography (TTE) confirms the diagnosis and determines the degree of severity; this test also describes biventricular geometry and function, detects other valvular diseases, as well as aortic and pericardial pathology; and finally provides prognostic information ^2^^,^^3^.

The usefulness of TEE is relevant for the study of concomitant mitral pathology when TTE is inconclusive. However, its greatest relevance is in situations of AS gradient-area discrepancies, where it provides useful information to specify the degree of severity (diameter/planimetry of the left ventricular outflow tract, aortic planimetry, and gradients in trans-gastric view) ^2^^,^^31^.

#### Aortic plaques

TEE is the imaging modality of choice to detect aortic plaques and characterize their morpho-structure ^9^^,^^18^^,^^32^^,^^33^. The main test quality is the high spatial resolution of the acquired image, thanks to the use of a high-frequency transducer and the esophageal proximity to the aortic arch ^13^^,^^34^.

Aortic plaques are strictly defined in this modality as discrete protrusions greater than or equal to 2 mm in thickness ^13^^,^^25^^,^^35^. Katz used grades to classify atherosclerosis in the aortic arch by TEE, in which he identified a subset of patients with a high risk of stroke when undergoing cardiopulmonary bypass: Grade 1: Apparently normal aortic arch; Grade 2: Extensive intimate thickening; Grade 3: Sessile atheroma protruding into the aorta <5mm; Grade 4: Protruding atheroma >5mm and Grade 5: Mobile atheroma ^36^.

**Figure 1:**
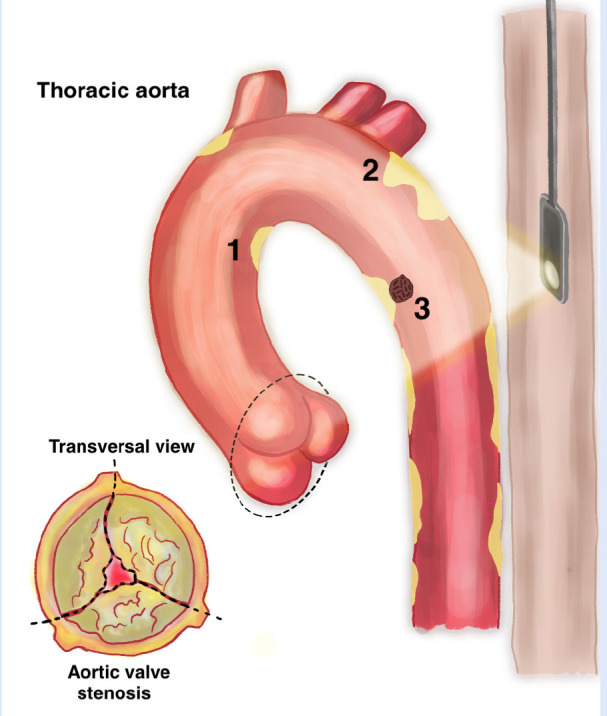
Transesophageal echocardiography (TEE) aortic atheroma tous plaques in aortic stenosis. Degenerative aortic stenosis is asso ciated with atherosclerotic thoracic aorta, and both pathologies are studied by TEE. The three types of complex plaques are shown: plaque ≥ 4mm (1), ulcerated plaque (2), and plaque with mobile elements ^(3)^.

Furthermore, the term complex aortic plaque refers to plaques with any of the following characteristics: thickness ≥ 4 mm, ulcerated or with mobile thrombi ^9^^,^^37^**(**Figure 1**)**, which are independently associated with stroke in patients with AS ^25^.

Concerning the performance of TEE in detecting aortic plaques, previous studies reported a sensitivity of 93% and a specificity of 82%, while the positive and negative predictive values were 88% and 90%, respectively. It is worth mentioning that these data are from 20 years ago ^38^, so it is possible that with current machines that have greater resolution power and new options such as 3D, diagnostic performance will be greater. Furthermore, it should be noted that TEE presents excellent intra- and extra-observer reproducibility (97% and 98%) ^29^.

The limitations of the test represent the impossibility of exploring the distal third of the ascending thoracic aorta ^9^^,^^21^^,^^29^, and the discomfort caused by the probe in the upper esophageal position during the study of the aortic arch.

### 3D TEE in atheromatous plaques

The multiple 3D TEE tools provide accurate, detailed, and complete information on the aortic plaques. Simultaneous double image or multiplanar reconstruction from an acquired volume allows us to have better precision of the dimensions of the plaques than a 2D study (greater height with a variation of approximately 0.1 cm +/- 0.06 cm (p<0.05)) ^39^. Qualitatively, the transverse and longitudinal planes derived from 3D provide images of high anatomical detail, which allows determining the “soft” content of the plaque, the contour of the fibrous capsule, the presence of the ulcer, or mobile elements of the plaque ^40^^-^^43^. Finally, the transillumination tool displays an image of the aortic plaque and its morpho-structural characteristics with the highest level of realism that any imaging technique can currently achieve ^44^.

### Tomography and magnetic resonance in aortic plaques

Computed tomography angiography (CTA) has a lower capacity to detect soft plaques (non-calcium) than TEE, so it has a sensitivity of 52.6% and a specificity of 92% ^45^. However, the total plaque burden score in the ascending aorta estimated by tomography represents a relevant prognostic role in patients undergoing cardiothoracic surgery ^46^. The earliest diagnosis of aortic atherosclerosis with CTA compared to TEE provides more time for decision-making. An additional advantage of this technique and MRI compared to echocardiography is the ability to scan the entire thoracic aorta, while the use of nephrotoxic contrast and radiation exposure represent the weaknesses of this modality.

Magnetic resonance imaging (MRI) allows the aortic plaque to be perfectly characterized, including the fibrous layer, lipid core, and thrombus ^47^^,^^48^. In T2 sequences, the fibrous layer and the thrombus present a high signal, while the lipid nucleus emits a low signal intensity ^47^. Regarding reproducibility, this modality has excellent intra- and inter-observer variability for the detection of aortic plaque ^49^. A recent study using 3D multi-contrast CMR determined the relationship of plaques ≥4 mm thick in the aortic arch and descending aorta with stroke ^50^. Unfortunately, availability and estimated price limit its routine practice **(**Table 1**)**.

**Table 1:** Comparison of strengths and weaknesses of imaging modalities for aortic plaques.

	Strengths	Weaknesses
TEE 2D	-Gold standard to detect aortic plaques. -High spatial and temporal resolution images. -Excellent intra- and extra-observer reproducibility. -Provides complete echocardiographic information.	-Distal third of ascending aorta is blind spot. -Discomfort of the procedure.
TEE 3D	-Optimal morphostructural characterization of aortic plaques. -Greater precision in measuring aortic plaque -Realistic images. -Provides complete echocardiographic information	- Software availability. -The same as the 2D TEE.
CTA	-High specificity to detect aortic plaques. -Useful in detecting calcified plaques. -Prognostic role in cardiothoracic surgery. -Complete thoracic aorta exploration.	-Low sensitivity to detect complex plaques. -Use of contrast. - Radiation exposure.
MRI	-Morphological characterization of the plaque. -Complete thoracic aorta exploration. - Excellent intra- and extra-observer reproducibility.	-Low availability of the technique. -Use of contrast.

TEE: transesophageal echocardiography. CTA: Computed tomography angiography. MRI: Magnetic resonance imaging

**Figure 2:**
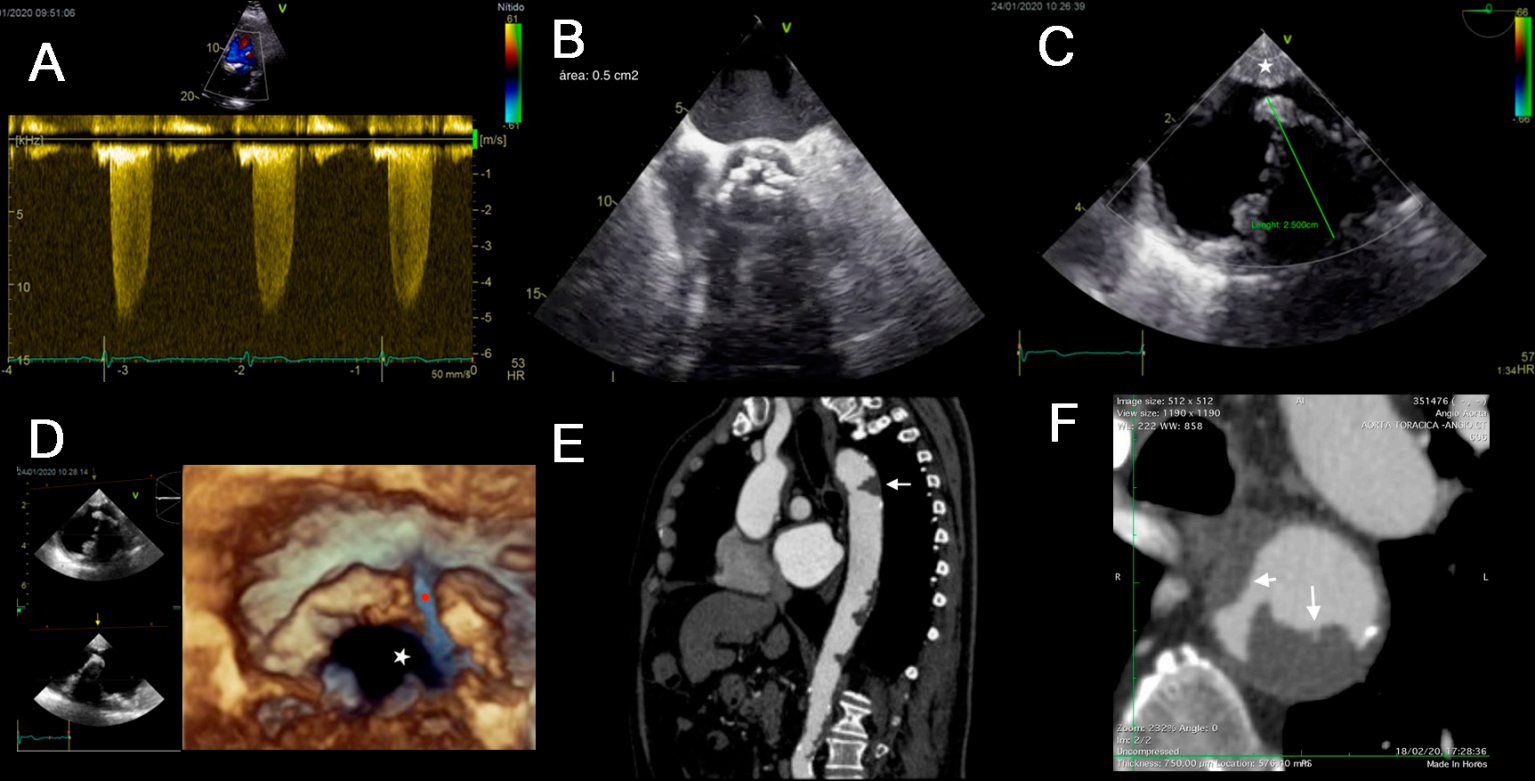
Aortic stenosis, ulcerated plaque, and thrombus

**Figure 3:**
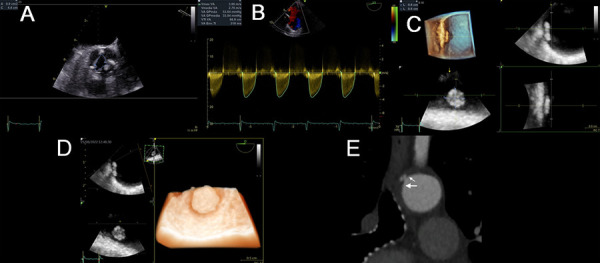
Aortic stenosis and occluder-like plaque

**Figure 4:**
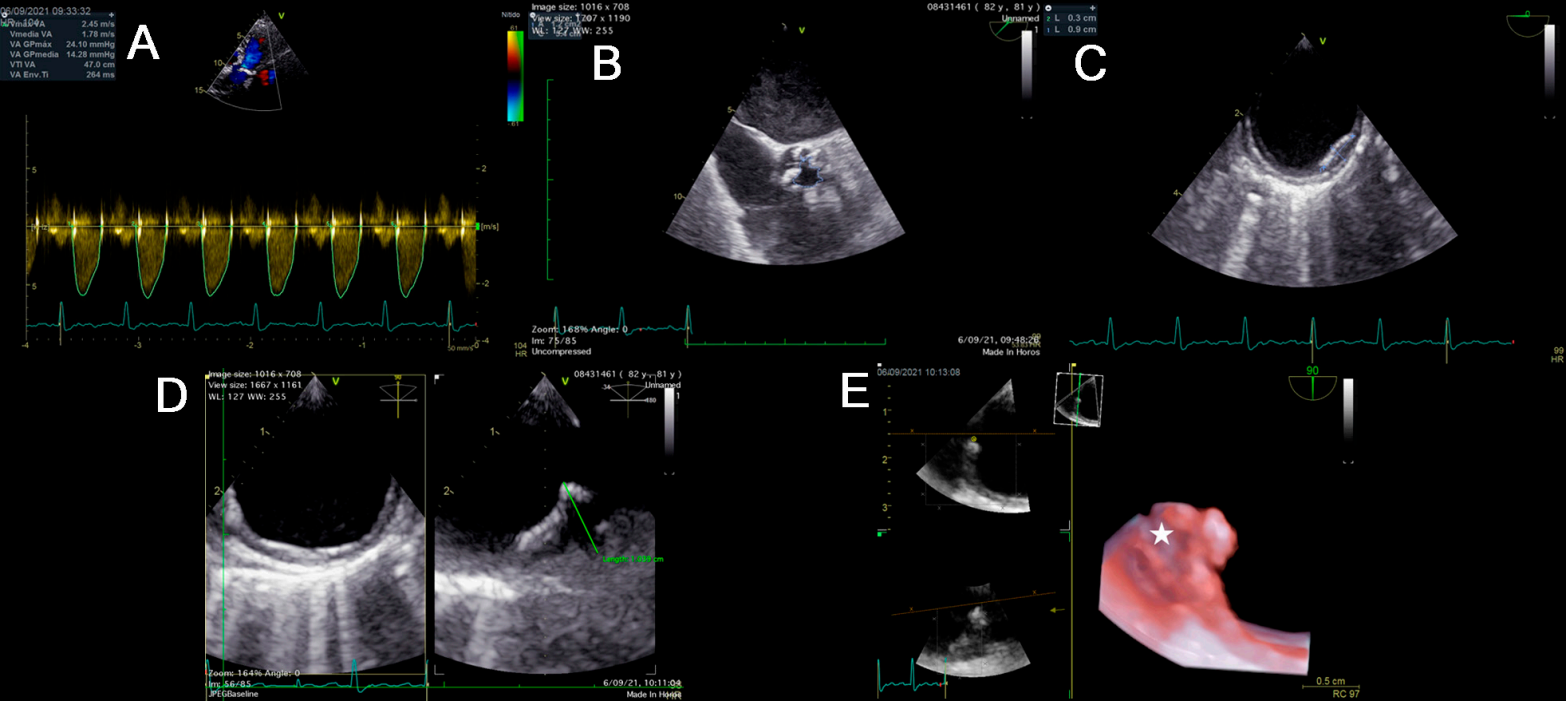
Aortic stenosis and complex plaque

**Figure 5:**
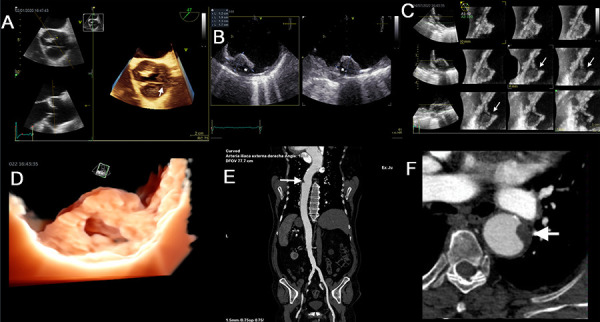
Bicuspid aortic stenosis and ulcerated plaque in maximum resolution

**Figure 6:**
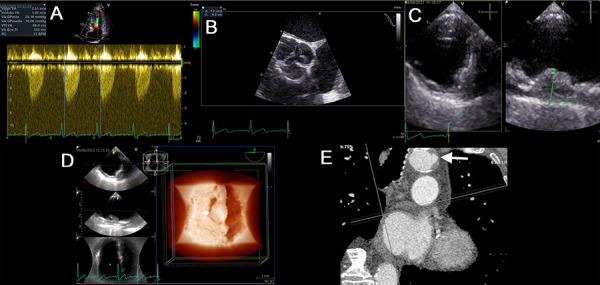
Aortic stenosis and complex plaque

We attach five illustrated cases, which demonstrate the usefulness of transesophageal echocardiography and its three-dimensional tool in the study of aortic plaques **(**Figures 2**-6)** (Videos 1-16).

An 86-year-old man with dizziness and progressive dyspnea for 1 year. History of arterial hypertension. **(A)** TTE, maximum transaortic velocity: 4.5 m/s. **(B)** TEE, area by planimetry of 0.5 cm2 (video 1). **(C)** TEE, at the proximal descending aorta, a large intraluminal thrombus (25 mm) with a wedge shape was found (video 2), and a minor wall plaque (white star). **(D)** 3D-TEE, complicated plaque (white star) with a medial fracture (red dot) inside the thrombus (video 3). **(E)** CTA, thrombosed plaque 5cm distal to the arch (white arrow). **(F)** transverse plane shows its morphology and intraluminal magnitude, the presence of irregularities on the surface (large white arrow), and the minor plaque (small white arrow).

An 86-year-old woman with a new syncopal event, chest pain on exertion, and dyspnea on mild exertion. Medical history of arterial hyperten sion and diabetes mellitus. **(A)** TEE, area by planimetry of the aortic valve: 0.9 cm2 (video 4). **(B)** maximum velocity, 3.6 m/s from deep transgas tric view at 13°. **(C)** Multiplanar reconstruction of an acquired volume of the descending aorta shows a 6-mm-diameter spherical wall plaque (video 5). **(D)** Volume rendering with the transillumination technique, an uncomplicated wall plaque with high realism (video 6). **(E)** The CTA, the location and morphology of the plaque (large arrow), and an adjacent plaque (small arrow).

An 82-year-old woman with dyspnea on moderate exertion. History of arterial hypertension, persistent atrial fibrillation with anticoagulation, and chronic kidney disease. **(A)** TTE, maximum transaortic velocity of 2.45 m/s. **(B)** TEE, area of aortic stenosis of 1.2 cm2 (Video 7) by planimetry. **(C)** A 3 mm-thick mural plaque at the mid-esophageal level. **(D)** A 10 mm-thick complex plaque at the upper esophageal level (video 8). **(E)** volume rendering shows a content of lower echogenicity corresponding to lipid material (white star) (video 9).

An 81-year-old man with dyspnea on moderate exertion and chest pain. History of aortic valve disease. **(A)** Volume rendering, bicuspid aortic valve with the fusion of the right and left coronary leaflets (white arrow), stenosis area of 0.9 cm2 by planimetry (video 10). **(B)** TEE, complex ulcerated plaque 13 mm thick in the thoracic aorta (video 11). **(C)** The multiplane tool illustrates the plaque and confirms in great detail the level of ulceration (white arrows) (video 12). **(D)** Transillumination volume rendering of the plaque, plaque ulceration with maximum realism (video 13). **(E)** The CTA of the thoracic aorta, location of the complex plaque (arrow). **(F)** and the transverse plane shows morphology and intraluminal magnitude (arrow).

A 74-year-old man with dyspnea on moderate exertion and leg swelling. History of arterial hypertension, diabetes, dyslipidemia, obesity, and peripheral arterial insufficiency. **(A)** TTE, transaortic velocity of 2.5 m/s. **(B)** TEE, area of aortic stenosis at 1.2 cm2 (video 14). **(C)** At the upper eso phageal level, a complex 11 mm plaque with irregular edges was evidenced (video 15). **(D)** Transillumination volume rendering of a complex bulging plaque from a long-axis view of the aorta (arrow) (video 16). **(E)** The CTA of the thoracic aorta in the transverse plane shows irregular morphology and intraluminal occupation of the complex plaque (white arrow).

## Conclusion

The coexistence of severe AS and complex plaques in the thoracic aorta is based on the fact that they share pathogenic pathways in their origins. This association is frequent and increases the risk of stroke, furthermore, AS can predict the presence of complex plaques, and conversely, these can predict the rapid progression of AS.

TEE is the test of choice for detecting thoracic aortic plaques and determining complexity characteristics. The 3D tool is more accurate in determining the dimensions and ulceration of the atheroma than 2D. The guidelines do not recommend routine TEE in patients with AS, however, due to the evidence presented, performing TEE to detect complex plaques in the thoracic aorta could be recommended, before percutaneous or surgical replacement of the aortic valve. 
